# Distinct functional levels of human voice processing in the auditory cortex

**DOI:** 10.1093/cercor/bhac128

**Published:** 2022-03-26

**Authors:** Matthias Staib, Sascha Frühholz

**Affiliations:** Cognitive and Affective Neuroscience Unit, University of Zurich, 8050 Zurich, Switzerland; Cognitive and Affective Neuroscience Unit, University of Zurich, 8050 Zurich, Switzerland; Neuroscience Center Zurich, University of Zurich and ETH Zurich, 8050 Zurich, Switzerland; Department of Psychology, University of Oslo, 0373 Oslo, Norway; Center for the Interdisciplinary Study of Language Evolution (ISLE), University of Zurich, 8050 Zurich, Switzerland

**Keywords:** voice, voice area, auditory cortex, MVPA, fMRI

## Abstract

Voice signaling is integral to human communication, and a cortical voice area seemed to support the discrimination of voices from other auditory objects. This large cortical voice area in the auditory cortex (AC) was suggested to process voices selectively, but its functional differentiation remained elusive. We used neuroimaging while humans processed voices and nonvoice sounds, and artificial sounds that mimicked certain voice sound features. First and surprisingly, specific auditory cortical voice processing beyond basic acoustic sound analyses is only supported by a very small portion of the originally described voice area in higher-order AC located centrally in superior Te3. Second, besides this core voice processing area, large parts of the remaining voice area in low- and higher-order AC only accessorily process voices and might primarily pick up nonspecific psychoacoustic differences between voices and nonvoices. Third, a specific subfield of low-order AC seems to specifically decode acoustic sound features that are relevant but not exclusive for voice detection. Taken together, the previously defined voice area might have been overestimated since cortical support for human voice processing seems rather restricted. Cortical voice processing also seems to be functionally more diverse and embedded in broader functional principles of the human auditory system.

## Introduction

Previous research in human (Belin et al. 2000; Pernet et al. 2015) and nonhuman primates (Petkov et al. 2008; Sadagopan et al. 2015) proposed a largely extended region in the AC, referred to as “voice area” (VA) (Yovel and Belin 2013; Pernet et al. 2015), that covers approximately 70%–80% of the human AC, including primary (Te1.0–1.2), secondary (planum temporale, PTe), and higher-level AC subregions (Te3) (Pernet et al. 2015; Agus et al. 2017; Aglieri et al. 2018). This VA was proposed to respond to voices with high sensitivity. Besides its large spatial extent comprising many subareas of AC, the VA seems to also comprise 3 specific regional peaks or “patches” of activations along the posterior-to-anterior axis of the superior temporal cortex (STC) (Pernet et al. 2015; Belin et al. 2018). These patches were proposed to specifically differentiate between voice signals and other auditory objects beyond any basic acoustic differences between these sound categories (Belin et al. 2000; Agus et al. 2017). However, a detailed and differential functional description of the VA in general and specifically of the voice patches for generic voice processing is largely missing (Staib and Frühholz 2020). In terms of such potential functions of different VA subareas, some anterior STC patches might be involved in voice identity decoding (Belin and Zatorre 2003; Kriegstein and Giraud 2004), anterior and middle STC patches might do some voice-specific acoustical processing (Charest et al. 2013; Latinus et al. 2013; Ahrens et al. 2014), and posterior STC patches might integrate auditory and visual information during voice recognition (Watson et al. 2014; Pernet et al. 2015). However, beyond this incoherent evidence for a sensitivity of some VA subareas for decoding types of voice information, a clearer functional description of the entire VA and its patches is missing, including its capability to generically discriminate voice signals from other auditory signals.

The notion of high sensitivity or even selectivity for voice processing across the entire VA was derived from 3 strands of evidence. First, the VA was discovered across several mammalian species, including primates and dogs, responding to con- and hetero-specific vocalizations (Belin et al. 2000; Petkov et al. 2008; Andics et al. 2014; Sadagopan et al. 2015). Second, given the frequent observation for face-selective regions in the primate visual system (Kanwisher and Yovel 2006), a parallel functional system was assumed to also exist in the AC for voice processing (Yovel and Belin 2013; Belin et al. 2018). Third, recent reports comparing generic voice processing to specific acoustically matched control sounds found a sensitivity of the VA for real voices. For example, a previous study (Agus et al. 2017) has shown that the right VA still reliably distinguishes between voice and nonvoice sounds when controlling for acoustic features, such as pitch, harmonic-to-noise ratio, temporal rate, and spectral scale of the sounds. Especially the activation in the left VA did not show a significant difference when controlling for acoustic differences between voice and nonvoice sounds. It was therefore proposed that the VA is selectively and rather uniformly sensitive to human voices compared to other nonvoice auditory objects beyond basic acoustic differences of voice and nonvoice sound sets used to define the VA (Yovel and Belin 2013; Agus et al. 2017; Belin et al. 2018). Previous studies demonstrating the nonsensitivity of the VA to acoustic differences between voice and nonvoices, however, have some shortcomings, such as rather arbitrary selections of features for experimental testing and an insufficient explanation of contradictory findings concerning voice processing in the left VA (Agus et al. 2017).

Using a more direct and precise experimental approach, we have also recently shown that the selectivity of the VA for voice processing seems to be largely overestimated, given that the entire VA can also respond with the same activity pattern to nonvoice textural noise patterns that are typically observed in voice processing (Staib and Frühholz 2020). Many subareas of the VA, especially in low- and higher-order AC, also responded nonselectively to acoustic features in any sound, both contained in voice and nonvoice sounds. Thus, the VA also seems to largely respond to nonvoice noise patterns and acoustic features with a similar cortical activity pattern, and this similarity in the cortical processing of voice and nonvoice sounds seems to point to some more basic neural mechanisms for auditory recognition in large portions of the VA beyond its assumed selectivity for voice processing. While the concept of selectivity for voice processing in the entire VA (Yovel and Belin 2013; Belin et al. 2018) might thus not be adequate to describe the mechanisms for voice processing in the VA (Staib and Frühholz 2020), there might be however different levels of (nonexclusive) neural sensitivity for voice stimuli and voice features at different sound processing levels of the AC from acoustic sound analysis to acoustic object classifications.

Accordingly, another missing part of these previous reports is the lack of a functional parcellation of the VA (Belin et al. 2018). While the subdivision of the VA into 3 patches seemingly parallels sensory processing observed for face perception in the visual system (Yovel and Belin 2013), where the so-called face patches are distinct clusters with differential functional profiles (Grill-Spector et al. 2006; Tsao et al. 2008), such a differential functional account is surprisingly missing for VA subpatches (Belin et al. 2018). This lack of a functional parcellation of the VA so far might indeed reflect a rather uniform functional and neural nature of VA despite diverse acoustic stimulations (Agus et al. 2017). To determine a possible functional parcellation of the VA, it might seem necessary to apply experimental designs that more directly account for the differential working principles of the human auditory system and the specific psychoacoustic properties of voice sounds. From these working principles and psychoacoustic effects, it seems possible that a proportion of the noncore and accessory VA primarily responds to the acoustic features and acoustic patterns inherent to but not exclusive for voice sounds, rendering these accessory VA areas more responsible for an acoustic sound pattern analysis for various sounds rather than for generic voice processing. Complementarily, core VA areas might generically differentiate voice from nonvoice based on a perceptual or even socially relevant difference between these sound categories. This approach would overall redefine the broader VA region as a local functional network in AC comprising subregions that have a differential functional contribution to voice processing.

**Fig. 1:**
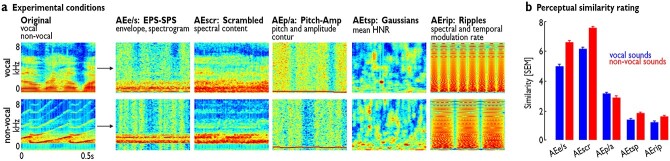
Original voice and nonvoice sounds and examples of their acoustic equivalents (AEs). a) Spectrograms of examples of original voice and nonvoice sounds and the 5 AEs that preserve certain acoustic features of the original sounds. The top row shows an original voice sounds and its corresponding AEs, whereas the bottom row shows an example nonvoice sounds and its AEs. b) Average perceptual similarity ratings comparing each original sound to its various AEs. The similarity rating was performed on a 10-point scale ranging from not similar at all (“0”) to highly similar (“10”).

We tested this hypothesis of a functional segregation of the VA into core voice processing subareas and more voice-unspecific incidental subareas by presenting standard voices and nonvoices sets (Belin et al. 2000; Capilla et al. 2013) to human volunteers, as well as 5 matched and carefully selected sets of acoustic equivalents (AEs) that were generated from the original sounds. These AEs had decreasing acoustic and perceptual similarities to human voices and nonvoice sounds but preserved selected features of the original sound sets (Fig. 1) such as (i) the spectral content (Ellis 2010; Overath et al. 2015), (ii) the general temporal and spectral envelope (Fukushima et al. 2014), (iii) the pitch and amplitude contour (Grandjean et al. 2005), (iv) the harmonic-to-noise ratio (HNR) (Lewis et al. 2009), or (v) the dynamic spectrotemporal modulation rate (Elliott and Theunissen 2009). These 5 types of AEs were chosen as they represent important acoustic features that support the detection and classification of a voice signal as an auditory object to various degrees (Lewis et al. 2009; Latinus and Belin 2011; Frühholz et al. 2018). And these AEs have been previously shown to elicit activity in AC subregions that also overlap with the VA without being perceived as a sound of vocal origin (Lewis et al. 2009; Schönwiesner and Zatorre 2009; Fukushima et al. 2014). We expected that core subregions reflecting generic voice processing areas of the VA should show a strong response to voices compared to nonvoices over and above any AE-based brain activity, indicating their sensitivity to the perceptual distinctiveness of voice sounds. These core VAs are expected to be localized in higher-level AC as a potential neural site for auditory objects classification (Kumar et al. 2007). Our study thus followed 2 major scientific approaches: first, using this set of AEs, we aimed to investigate a functional distinction in the general VA along acoustic and perceptual features of the VA that would allow separation into core and accessory VA subregions. Second, using 2 complementary analysis types in the form of a univariate and a multivariate analysis approach, we aimed to define subregions of the VA that are generic to voice processing and voice feature decoding, respectively.

## Materials and methods

### Participants

Twenty-five volunteers (13 female, mean age 25.3 years, SD 3.97) participated in the functional magnetic resonance imaging (fMRI) experiment. Inclusion criteria were normal or corrected-to-normal vision and no history of neurological or psychiatric disorders. All participants gave written informed consent and were financially reimbursed for participation. The study was approved by the cantonal ethics committee of the Canton Zurich (Switzerland).

### Stimuli

The set of natural sounds consisted of recordings of 70 vocalizations (speech and nonspeech) and 70 nonvocalizations (animal, natural, and artificial sounds) (Capilla et al. 2013) of 500 ms duration. As described in Capilla et al. (2013), vocal stimuli were recorded from 47 speakers (from babies to elderly people) and were either speech (nonwords) or nonspeech (laughs, sighs, and various onomatopoeia). Nonvocal stimuli consisted of sounds from nature (e.g. wind, streams), animals (cries, gallops), the human environment (cars, telephones, planes), or musical instruments (bells, harp, and instrumental orchestra). For each sound, 5 AEs were created (Fig. 1), from the categories (i) envelope/spectrum preserved sounds (AE_e/s_), (ii) scrambled sounds (AE_scr_), (iii) pitch/amplitude preserved sounds (AE_p/a_), (iv) ripple sounds (AE_rip_), and (v) textural sound patterns (AE_tsp_). AEs were designed such that their original sound identity was unintelligible by the listener, but each AE shared an important acoustic feature with the original sounds. Using these AEs, we aimed to determine the part of the original VA that also shares cortical processing for AEs that carry a certain acoustic feature as typical for voice sounds. These AEs were created both for the original voice and nonvoice sounds, and we kept this original assignment for the AEs, such that these AEs were individually defined as being derived from either a voice or nonvoice sound. Therefore, we performed the same contrasts on the AEs as for defining the VA with the standard contrast [voice > nonvoice].

Furthermore, the 5 AEs decreased in similarity to the original sounds as can be seen from the spectrograms (Fig. 1a) and the similarity ratings by the participants (Fig. 1b). This is different to one of very few other studies that are directly comparable with our approach investigating the influence of acoustic features on voice/nonvoice processing of the VA (Agus et al. 2017). These authors matched sounds of vowels sung by professional singers by pitch and HNR with tones played on different classical instruments and created “auditory chimeras” that have either the spectral profile of the voices and the temporal profile of the instrumental sounds or vice versa. However, even if this study also includes some acoustic parameters as modulators in their models, the auditory chimeras are by definition artificially created mixtures of voices and nonvoices. Furthermore, the authors admit to having controlled for some (e.g. pitch, HNR) but not all possible acoustic differences while creating matches between voices and nonvoices. Last but not least, the sung vowels taken as voice stimuli are a very selective sample of voice stimuli compared to the often used stimuli of the VA localizer (Belin et al. 2000; Capilla et al. 2013; Pernet et al. 2015) and include stimuli with language content. Another study by Norman-Haignere and McDermott (2018) compared neural responses in AC between natural auditory objects and matched synthetic sounds (i.e. matched according to a sensory model of neural auditory processing) and found similar neural responses in primary AC to both sound sets, but divergent responses in nonprimary AC. Nonprimary AC specifically showed some sensitivity for the processing of speech and music. While this study (Norman-Haignere and McDermott 2018) might have pointed to some specificity in the neural processing of speech as a specific voice stimulus, it does not provide evidence for the more basic mechanisms of the neural processing of (nonspeech) voice signal processing in general. Their study (Norman-Haignere and McDermott 2018), however, points to a valuable experimental approach of using matched synthetic sound to test AC mechanisms for sound processing. Synthetic AEs of individual sounds that preserve only one type of acoustic information from the original sounds offer a direct comparison between brain responses to the originals and their equivalents. The possibility to perform the same contrasts on AEs and original voice and nonvoice sounds is much more promising as an attempt to investigate acoustic processes by the temporal VA.

A detailed description of how the AE_e/s_ that preserve the envelope and the spectrogram of a sound were generated can be found elsewhere (Fukushima et al. 2014). Briefly, for AE_e/s_, we first calculated the amplitude of the Hilbert transform from the original stimulus. This amplitude envelope was then multiplied with a broadband white noise, resulting in sounds with the same flat spectral content. For spectrum preserved stimuli, broadband white noise with a duration of 500 ms was generated, and its amplitude in the Fourier domain was replaced by the average amplitude of the corresponding stimulus. The result was then transformed back to the time domain by the inverse Fourier transform. Finally, envelope and spectrum preserved stimuli were added together and normalized. The AE_e/s_ are AEs that preserved the general temporal and spectral envelope profile of the sounds, which could be one of the acoustic markers that distinguish voice from nonvoice sounds (Stanley 1931).

The sounds for the pitch and amplitude envelope preserving sounds AE_p/a_ were sounds combined out of sine-wave rectified pitch contour of the original sounds together with a broadband white-noise sound with an amplitude envelope derived from the original sounds. The modulated white-noise sound was overlaid with the sine-wave pitch contour sounds and played at the same time. These sounds preserved the pitch and intensity dynamics of the original sounds (Grandjean et al. 2005).

The scrambled acoustic equivalents AE_scr_ were created by scrambling each audio file by moving around short, overlapping windows within a local window, such that spectral content over longer time scales can be preserved, but structure at shorter timescales is removed. Here, the signal was first broken into multiple frequency bands, scrambled in each band separately, and finally recombined (ee.columbia.edu/∼dpwe/resources/matlab/scramble/) (Ellis 2010). These scrambling procedures preserved the overall spectral content of the sounds, but this content was shuffled in time to destroy the temporal dynamics of the original sounds.

The set of ripple sounds that were the basis of AE_rip_ was created using in-house Matlab code based on a previous description of ripple sounds (Schönwiesner and Zatorre 2009) and by selecting from 10,000 sounds with varying velocity (amplitude modulation), density (frequency modulation), and modulation depth. For each original sound, we selected the best matching ripple sound based on the highest similarity in acoustic features (mean and standard deviation of jitter, shimmer, and spectral flux features), extracted with the publicly available toolbox openSmile v2.3.0 (Eyben et al. 2013) and based on an automated selection algorithm.

The set of AE_tsp_ finally was generated with the Gaussian Sound Synthesis Toolbox (Version 1.1; mcdermottlab.mit.edu/downloads.html) (McDermott et al. 2011). First, 10'000 textural sound patterns (TSPs) of 500 ms duration were generated with frequency and temporal correlations between 0.01 and 2, respectively. For each TSP, the mean and standard deviation of the HNR (Eyben et al. 2016) was extracted. To find a matching AE_tsp_ for each original sound, the sound with the minimal Euclidean distance in a 2-dimensional acoustic space (based on the normalized HNR features) was selected. All sounds in this study had a 5 ms cosine rise/fall to avoid abrupt onset/offset of the sound and were normalized to 70 dB SPL.

### Experimental procedure

The sound presentation in the MRI scanner was spread over 8 runs, where each run lasted 315 s and contained 12 mini blocks with one stimulus type each; that is in one block, 9 sounds were presented from either the original sound sets or one of the 5 AE types (6 sound types in total), separately for voice and nonvoice stimuli. The order of mini blocks within each run was completely randomized, and the sounds selected for a mini block were also randomized in their order within the mini block. The 12 mini blocks within each run were composed of the 12 different sound categories, and thus each sound category appeared once in each run. The time between sounds within a mini block was jittered within 0.8–1.2 s, and the time without any sound between the mini blocks was 15 s. In 24 out of the 96 blocks (8 runs × 12 blocks) across the experiment, one single sound was played twice consecutively. Participants had to indicate whenever they detected a repetition of a sound with a button press at the end of the respective block. This repetition detection task ensured that participants showed the same level of listening engagement for all sound categories and mini blocks, and the repetition of all sound categories in each run and the randomization of mini blocks ensured that no block and run order effects appeared.

In a separate perceptual rating experiment, an independent sample (*n* = 26, 18 female, mean age 24.92 years, SD 4.45) was presented with pairs of an original sound and each of its corresponding AE in separate trials. This procedure was repeated for each of the original sounds (70 voices, 70 nonvoices) and its AEs, resulting in a total of 700 trials. Participants had to rate the perceived similarity on a discrete scale from 0 to 10 (0 = not similar at all, 10 = highly similar).

### Functional brain data acquisition

Functional brain data were recorded in a 3T-Philips Ingenia with a standard 32-channel head coil. High-resolution structural MRI was acquired by using T1-weighted scans (301 contiguous 1.2 mm slices, time repetition (TR)/time echo (TE) 1.96 s/3.71 ms, field of view 250 mm, in-plane resolution 1 × 1 mm). In each run, 197 functional whole-brain images were recorded by using a T2^*^-weighted echo-planar pulse sequence (TR 1.6 s, TE 30 ms, FA 82°; in-plane resolution 220 × 114.2 mm, voxel size 2.75 × 2.75 × 3.5 mm; 28 slices, slice gap 0.6 mm) covering the whole brain. For each participant, a whole-brain magnetic field mapping sequence (TR 30 ms, TEs 0.01/3.57 ms, FA 60°, voxel size 2.7 × 2.7 × 4 mm) was recorded to reduce image distortions from inhomogeneities in the magnetic field. Cardiac and respiratory variation was measured using the scanner inbuilt pulse oximeter and respiration belt.

### Brain data preprocessing

Preprocessing of fMRI data was performed by using standard procedures in the Statistical Parametric Mapping software (SPM12; Wellcome Trust Centre for Neuroimaging, London, UK; www.fil.ion.ucl.ac.uk/spm/software/spm12). Images were corrected for geometric distortions caused by susceptibility-induced field inhomogeneity (Cusack et al. 2003). A combined approach was used, which corrects for both static distortions and changes in these distortions from head motion (Andersson et al. 2001; Hutton et al. 2002). The static distortions were calculated for each subject from a B_0_ field map that was processed by using the FieldMap toolbox as implemented in SPM12. With these parameters, functional images were then realigned and unwarped, a procedure that allows the measured static distortions to be included in the estimation of distortion changes associated with head motion. Slice time correction was performed to correct for differences in acquisition time of individual brain slices. The motion-corrected images were then co-registered to the individual’s anatomical T1 image by using a 6-parameter rigid body transformation. Functional data were normalized from the participants’ space to the Montreal Neurological Institute (MNI) space based on the IXI549 dataset (brain-development.org/ixi-dataset/) using a unified segmentation procedure as implemented in the Computational Anatomy Toolbox (CAT12; www.neuro.uni-jena.de/cat/) with a 2 mm isotropic resolution. The deformation parameters were computed from normalizing the T1 images of each participant. For the region of interest (ROI)-based multivariate pattern analysis (MVPA) analysis in the native space of each participant, the inverse deformation parameters originating from the aforementioned segmentation procedure were used to warp standardized atlas-based brain maps into the space of each participant.

### Brain data analysis

After preprocessing, we performed 2 different types of analysis. First, we estimated the blood oxygen level-dependent (BOLD) amplitude in each voxel using a general linear model (GLM). This analysis was performed on functional data that were smoothed with an isotropic Gaussian kernel of full-width at half-maximum (FWHM) 8 mm and 4 mm in a subsequent replication analysis. Each block of 9 sounds was modeled with a boxcar function for the duration of the respective block, which was then convolved with the standard hemodynamic response function as implemented in SPM12. Additionally, a regressor to account for button presses was included, resulting in 13 regressors (6 sound sets × 2 vocalization conditions + button press), as well as 6 regressors of no interest for head motion that were estimated in the realignment step during preprocessing. Cardiac and respiratory variance was modeled with 18 RETROICOR regressors (Kasper et al. 2017) implemented in the PhysIO toolbox, which uses Fourier expansions for the estimated phases of cardiac pulsation (third order), respiration (fourth order), and cardiac-respiratory interactions (first order).

For the statistical group analysis, the linear contrasts for each of the 6 sound sets × 2 vocalization conditions across all 8 runs (i.e. one contrast image for each of the 12 conditions, separately for each participant) were defined and modeled in a 6 × 2

analysis of variance (ANOVA) in a full factorial analysis in SPM12 that can be combined with a conjunction analysis. A conjunction analysis identified voxels that are active during 2 or more conditions (Friston et al. 2005; Nichols et al. 2005) and was used here to estimate the spatial overlap of voice-sensitive activations across the original sound set and AEs. Next to this contrast-based GLM analysis, we performed an additional analysis for the purpose of potentially identifying “voice-selective” subregions of the AC as previously proposed (Belin et al. 2000; Perrodin et al. 2011). This additional analysis allowed to quantify neural voice selectivity within and across participants and to estimate a voxel-based measure of response magnitude to voice sounds and to all other sounds included in this experiment. For each participant, we therefore scaled the activation of each voxel by subtracting the beta estimates of the original nonvoice condition from each other condition and divided the result by the difference between the voice and nonvoice conditions. Using this scaling approach, we could quantify response magnitudes for all 12 experimental conditions relative to neural activity elicited by nonvoice (0%) and voice sounds (100%) as spanned by the original sound conditions. A voxel was labeled as “voice selective” if all 10 AE conditions showed a scaled magnitude lower than 33% (66% selectivity criterion) (Perrodin et al. 2011). This means a “voice selective” voxel would show a 3 times higher activation to the original voices than to any AE, relative to nonvoices. The binary voxel maps that distinguish selective and nonselective voxels were then summed up across participants (Supplementary Fig. S2).

Next to the GLM-based analysis, we performed a MVPA using a linear support vector machine (SVM) as implemented in The Decoding Toolbox, v3.96 (Hebart et al. 2015) (sites.google.com/site/tdtdecodingtoolbox/). This SVM was trained to separate 2 conditions, that is voice sounds from nonvoice sounds for the original sounds as well as for the AEs, respectively. The MVPA was performed on unsmoothed functional data in the participant’s native space, either using data from voxels in a ROI or in a searchlight approach (Kriegeskorte et al. 2006). For each classification scheme, the BOLD estimates from a separate GLM performed on unsmoothed data in native space served as data points. In an 8-fold cross-validation scheme with balanced data sets, a new model was trained on the BOLD estimate from 7 runs and tested on the remaining run, such that each data point was entered as test data point once. The final accuracy is the average of all 8 test predictions. After the MVPA calculations, voxel-wise decoding accuracy maps from the search light analysis were normalized to MNI space (i.e. using the deformation parameters as they originated from the segmentation approach) and smoothed with an isotropic Gaussian kernel of FWHM 8 mm. The group-level statistical analysis was performed as a GLM analysis using a one-sample *t*-test scheme for each cross-validation approach (i.e. 6 MVPA schemes, based on the 6 major sound categories) and cross-classification SVM approach (i.e. 5 MVPA schemes, based on the cross-classification between original sounds and the AEs) across experimental conditions.

For visualization purposes, we mapped the thresholded statistical maps that resulted from the various GLM analyses to a surface representation of the human brain using the CAT12 toolbox. Specifically, the statistical maps were resampled to a 164 k surface mesh according to a FreeSurfer surface template and were rendered on an inflated version of this surface template.

### ROI analysis

To further test how much information certain functional and anatomical subregions of the auditory cortex (AC) carry to distinguish original voice from nonvoice sound as well as for the AEs, we included both anatomically and functionally defined ROIs. For anatomically defined ROIs, we used atlas-based maps (Eickhoff et al. 2005), including primary (Te1.0, Te1.1, Te1.2), secondary (BA42), and higher-level AC regions (Te3), that were warped into the native space of each participant based on the deformation parameters obtained during preprocessing.

Three functionally defined ROIs were defined per hemisphere representing the 3 patches that are at the center of the VA and proposed to be highly sensitive to voice sounds (Pernet et al. 2015). To create the 3 ROIs specific to our sample, we followed a procedure described elsewhere (Aglieri et al. 2018). For each participant, the 10 peak locations of the contrast [voice > nonvoice] for the original sounds within bilateral AC were identified. The search space for these peak locations was constrained to the group-level activation map resulting from the contrast [voice > nonvoice] with a significance threshold of *P* < 0.001 (uncorrected). The center of each patch (anterior, mid, posterior) was then defined by the location nearest to the respective coordinate reported in Pernet et al. (2015). If the coordinate could not be identified or were closer to each other than 10 mm, the coordinates from Pernet et al. (2015) were used instead. Around each peak, a sphere with a 5 mm radius was constructed that included the voxels of the ROI.

### SVM trained on acoustic features

In addition to the MVPA approach on the brain data**,** we classified voice v**ersu**s nonvoice original and AE sounds based on the acoustics and compared the classification accuracies for the different subsets of sounds (original and **5** AEs). Here we first extracted 88 standard acoustic features (Eyben et al. 2016) using the openSMILE toolbox (audeering.com/opensmile/) and second trained an SVM (Cortes and Vapnik 1995) algorithm to acoustically discriminate original voice from nonvoice sounds as well as voice-based and nonvoice-based AEs with each class (cross-validation). The SVM is a binary classifier and used a 1-vs-1 scheme as implemented by MATLAB’s fitecoc function (MATLAB, version 2018a). The SVM parameters were set to the MATLAB default values and the kernel function was a third-order polynomial. In a next step**,** we also applied a cross-classification approach by training the SVM on the original sounds and tested the SVM on each of the AEs.

## Results

### AEs differentially resemble original voice and nonvoice sounds

Our goal was to compartmentalize the activation profile of the entire VA during voice perception into core and accessory voice processing subareas by using a selection of AEs that varied in their acoustic and perceptual similarity (or distance) to original voice and nonvoice sounds that are frequently used to define the VA (Belin et al. 2000; Capilla et al. 2013; Pernet et al. 2015). AEs were derived from the original sounds or were synthesized based on central acoustic features of the original sounds. We created 5 different AEs (Fig. 1a): AE_scr_ were scrambled versions of the original sounds by temporally shuffling time intervals and they preserved the overall spherical content (Overath et al. 2015); AE_e/s_ were synthesized sounds based on the mean spectral (envelope preserving sounds) and temporal profile (spectrum preserving sounds) of the original sounds (Fukushima et al. 2014); AE_p/a_ were synthesized sounds of a time-varying sine-wave rectified from the pitch contour combined with white noise that was amplitude modulated by the intensity envelope (Grandjean et al. 2005); AE_tsp_ were TSPs based on a Gaussian-derived spectrum that preserved the HNR of the original sounds (Lewis et al. 2009); and AE_rip_ were ripple sounds that resembled the spectrotemporal modulation rate of the original sounds (Elliott and Theunissen 2009; Schönwiesner and Zatorre 2009).

We first rated the perceptual similarity of the AEs to the original sounds by an independent sample of volunteers (*n* = 26, 18 female, mean age 24.92, SD 4.45). AEs based on nonvoice sounds were rated more similar to the original sounds than voice sounds [repeated measures ANOVA (rmANOVA), within factors were “category” (voice, nonvoice) and “modality” (5 AEs); *F*_1,25_ = 43.232, *P* < 0.001]. Across the AEs, we also found a significant difference (*F*_4,100_ = 210.980, *P* < 0.001), with differences between all AEs (all *P*’s < 0.001) except for AE_tsp_ and AE_rip_ showing no difference (*P* = 0.131). Based on a significant sound × AEs interaction (*F*_4,100_ = 45.375, *P* < 0.001), we found differences between AEs based on voices or nonvoices origin for all AE categories (all *P*’s < 0.001) except for the AE_p/a_ category (*P* = 0.067) (Fig. 1b).

### Sensitivity of the VA subareas to nonvoice acoustic sound patterns

We used functional neuroimaging in humans, while they listened to the original voice and nonvoice sounds as well as to the 5 AEs and performed an incidental 1-back detection task. If a sound was repeated within a block consisting of 9 sounds, participants had to press a button at the end of the block (see Methods). The participant’s correct positive rate was 70.83% (rate at which a repetition was discovered, given the presence of a repetition in a mini block). Conversely, in 22.67% of mini blocks without a repetition, participants falsely reported a repetition.

**Fig. 2:**
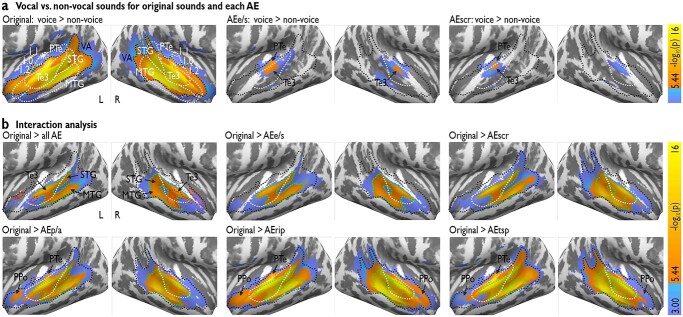
Spatial extent of the VA in bilateral AC. a) Voice compared to nonvoice sounds (left panel), and the same contrasts performed for the AEs. Blue activations *P* < 0.001 (uncorrected); orange activations *P* < 0.05 [family-wise error (FWE)-corrected]. Black dashed line denotes significant *P* < 0.05 (FWE) voxels from the original sound contrast, indicating that all significant voxels of AEs are contained within the original VA. White outlines indicate anatomical subparts of the AC, especially primary AC (Te1.0–1.2), secondary AC (PTe), and higher-level AC (Te3). b) The left upper panel shows voxels resulting from the conjunction analysis for which the contrast [voice > nonvoice] exceeds all the analogous contrasts performed on AEs (green dashed line). The individual interaction analyses compare the [voice > nonvoice] contrasts for original sounds against the same contrast in each of the AEs separately (shown in the mid and right upper and all lower panels). L, left; R, right, Te, temporal regions.

In the first set of MRI analyses, we used a univariate GLM approach to define both the general VA and the presumably core VA for voice processing. To define, first, the commonly reported general VA, we contrasted neural activations for original voice against nonvoice sounds and found largely extended bilateral AC activations centered on the STC [superior temporal gyrus (STG) and middle temporal gyrus (MTG)] and especially on the higher-level auditory region of Te3 (left *n* = 1,653 voxels, right *n* = 1,572 voxels). This activation also extended into primary (Te1.1) and secondary AC (PTe) (Fig. 2a, left panel). This broadly extended activation pattern in AC is commonly found in many studies for defining the VA (Pernet et al. 2015; Frühholz et al. 2020). A similar result of neural activity for original voices contrasted against original nonvoice sounds can also be found with a lower smoothing kernel applied to the functional data (Supplementary Fig. S1a). The latter also shows a broad extension of functional activity in the anterior-to-posterior direction in AC, as well as functional peaks located both in superior and MTG and in superior temporal sulcus (STS).

Second, we computed an analogous analysis for all AEs, contrasting the AEs of voices and nonvoices. Our hypothesis was that the AEs elicit activity in subareas of VA, thereby sharing a common activation profile with the original sounds. This would indicate that these areas do not respond selectively to voices but that they share some functional processing properties across acoustically equivalent sounds. For AE_e/s_ and AE_scr_, we indeed found activity in the AC centered in mid-superior Te3, but with a less spatial extent than the original VA. Notably, all significant voxels for this comparison within AE_e/s_ and within AE_scr_ were contained within the active cluster of the VA derived from the original sounds (Fig. 2a, black outlines). No other voice against nonvoice sounds for the other AEs revealed any significant activity in AC. Furthermore, for none of the sound sets (original or AEs) did any voxels show higher activation for nonvoices compared to voices.

Third, to obtain a refined estimation of the VA’s spatial extent, we next identified voxels for which the response to original voices compared to nonvoices exceeds the equivalent comparison in any of the AEs, which would point to the presumable core VA based on the GLM approach (Fig. 2b). For each AE, we computed the interaction contrast [(ORIGvoice > ORIGnonvoice) minus (AEvoice > AEnonvoice)] and combined them in a conjunction analysis that returns only those voxels where the original VA definition produces a higher response to voices than for all AEs. Figure 2b (upper left panel) shows a comparison between the VA activation based on the original contrast (black outline, Fig. 2a), overlaid on the refined area defined by the conjunction analysis (see also Supplementary Fig. S1b, which replicates this analysis with a lower smoothing kernel of the functional data). The refined VA contains significant voxels that were located in Te3 with extension into posterior STC (left *n* = 549 voxels, right *n* = 480 voxels), which corresponds to 33.2% (left) and 30.5% (right) of the size of the original VA activation (left *n* = 1653 voxels, right *n* = 1572 voxels), respectively. Notably, the refined VA does not exclusively overlap with the highest activation of the original VA, and 25% of it also includes lower active voxels of the original VA.

Besides this conjunction analysis of the interactions across AEs, the interaction contrasts are individually reported to allow a comparison between the original sounds with each AE separately (Fig. 2b, upper mid, right, and lower panels). For these individual interaction analyses, we found an overall increase of the spatial extent of the refined VA into anterior and posterior AC for AE_e/s_, AE_scr_, AE_p/a_, AE_rip_, and finally AE_tsp_. The interaction analysis for AE_tsp_ did cover large parts of the original VA but still did not fully cover the original VA, especially in the anterior STC and the planum polare (PPo).

Fourth, as a control analysis, we aimed to identify voxels that could exhibit a higher voice sensitivity for a given set of AEs than for the original sounds. For this, the reversed interaction between sound sets and stimulus type (i.e. [AEvoice > AEnonvoice] minus [ORIGvoice>ORIGnonvoice]) was computed, but no voxels responded more strongly to any AE than to the original sounds. We also repeated the interaction analysis as described above using a 4-mm FWHM smoothing kernel, replicating the result patterns as observed with an 8-mm FWHM smoothing kernel (Supplementary Fig. S1b).

Fifth and finally, we also aimed at determining of some subareas of the VA would show some kind of “voice-selectivity,” meaning that subareas would show largely increased neural activity to original voice sounds as compared to nonvoice sounds or any of the AEs sounds. For each voxel and participant, we scaled neural effects for all conditions from 0% to 100% based on neural activity for original nonvoice (set to “0%”) and original voice sounds (set to “100%”), respectively. If none of the 10 AEs conditions showed activity higher than 33% (66% selectivity criterion as often used in the literature; Perrodin et al. 2011), a voxel was labeled as “voice-selective” (labeled with “1,” otherwise “0”). These voxel-wise binary maps were summed across participants (Supplementary Fig. S2, Fig. 2b upper left panel). We found no subregions of the AC that showed strong indications of voice-selectivity, meaning the large majority of participants showed selectivity at a certain AC or specifically VA subarea. Some bilateral AC and VA subareas showed a tendency towards “voice-selectivity,” such that a maximum of a little more than half the participants (*n* = 13 out of 25 participants) showed consistent selectivity (Supplementary Fig. S2b). These regions were located in bilateral PPo and anterior STG, with the latter region partly overlapping with the afore defined core VA based on the interaction contrasts.

### Neural pattern specificity for voice processing in the VA

For the AC, it was recently shown that the response to voices compared to nonvoices manifests not only in the form of an increased voxel-wise BOLD amplitude, but it seems also encoded in the activation pattern of multiple neighboring voxels in the VA (Ahrens et al. 2014; Lee et al. 2015). Such multivoxel pattern activations can be detected with high sensitivity by multivariate analysis methods (Kriegeskorte et al. 2006), where a classification algorithm decodes local patterns of activations for voice versus nonvoice sounds. This multivariate analysis approach was the second major methodological approach beside the univariate GLM approach reported above. While the univariate GLM approach so far allowed to define the presumable core VA area, this MVPA approach is suitable to identify subregions of the VA that process acoustic information with a common mechanism across voices and AEs.

**Fig. 3:**
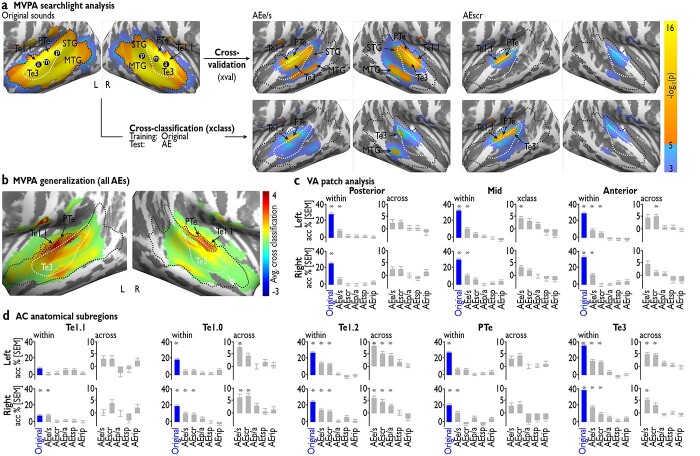
Results of the MVPA. a) The upper row shows cross-validated classifications of [voice versus nonvoice] for the original sounds and AEs. Black outlines are taken from the left-most panel (original sounds), indicating that all significant voxels within the AEs are contained within the original VA. The lower row shows the cross-classification approach with training on the original sounds and testing on the AEs. Significant voxel clusters from the cross-classification approach are marked with a green dashed line and displayed in the upper row. White outlines indicate Te3 as an anatomical landmark in the AC. The 3 VA patches [posterior (p), mid (m), anterior (a)] are taken from Pernet et al. (2015) and marked by black dots in each hemisphere. b) Average cross-classification results, highlighting that subareas of the original VA (black dashed line) in primary AC and PTe generalize the strongest from the original sounds to the AEs. c) Cross-validation accuracy (relative to the chance level of 50%) within each sound set (“within”) and cross-classification accuracy from the originals to AEs (“across”) for the 3 VA patches (posterior, mid, anterior). d) Cross-classification results equivalent to (c), but on anatomically defined subregions of AC instead of a searchlight approach. L, left; R, right; Te, temporal auditory regions. Asterisks indicate significant *P* < 0.05 (FDR corrected) above chance level classification.

To identify multivoxel patterns in our study, a SVM was trained to distinguish AC activation patterns of voices from nonvoices for the original sounds and equally for all AEs (Fig. 3a). This SVM analysis was performed on voxels within a sphere (radius 5 mm) that iteratively moved its center across all voxels in the form of a moving searchlight (Kriegeskorte et al. 2006). As shown in Fig. 3a (upper panels), the cross-validation procedure largely replicates the results from the above univariate GLM approach for the original sounds and AEs in the AC. Specifically, the MVPA succeeded in distinguishing original voice from nonvoice sounds in a bilateral and broadly extended cluster of voxels. This cluster contains a large portion of STC and reaches into PAC and PTe and seems larger in spatial extent (left *n* = 2,258 voxels, right *n* = 2,031 voxels) compared to the GLM results. Similarly, as for the GLM analysis, we then repeated the classification for each of the AEs. For AE_e/s_, we found a bilateral AC cluster that can distinguish the AEs of voices versus nonvoices in the superior part of Te3 (similar to the GLM contrast), with additional voxels found in MTG. For AE_scr_, we found a left AC cluster in mid-superior Te3 (again similar to the GLM contrast). For AE_p/a_, AE_rip_, and AE_tsp_, the cross-validation approach did not reveal significant voxels.

Given the AC overlap between voice detection for the original sounds and some of the AEs described above, we investigated the similarity between the response profiles of voice detection for the different levels of AEs compared to the original voices. This was done with a searchlight cross-classification approach, where a classifier was first trained to discriminate the original voices from nonvoices and then tested on each of the AEs to separate AE-based voice and nonvoice sounds (Fig. 3a, lower panels). For AE_e/s_ and AE_scr_, the classification was generalizable from the original sound sets, indicating that the same multivoxel representation that distinguishes the original voices from nonvoices is shared with some AEs. For AE_e/s_, we found a left AC cluster at the border of Te3 and Te1.1, and clusters in right posterior Te3 and in MTG. For AE_scr_, we found a similar left AC cluster located at the border of Te3 and Te1.1. For AE_p/a_, AE_rip_, and AE_tsp_, the cross-classification also did not reveal significant voxels, which could follow from an unsuccessful MVPA cross-validated classification of voices from nonvoices within these AEs (see above).

We then checked for AE_e/s_ and AE_scr_ whether all patterns that discriminate voice from nonvoice equivalents share this representation with the original sounds (Fig. 3a, green outlines). For AE_e/s_, all voxels with successful cross-classification are fully contained within the areas where the cross-validation-based distinction of voice versus nonvoice sounds within AE_e/s_ was significant. In turn, we found that for AE_scr_, local patches where the cross-classification was successful were not included in the within-AE_scr_ cross-validation results, indicating that the information representation of the original voices can be more robust and generalizable across AEs than the representation of scrambled voices.

To summarize the cross-classification results across all AEs, the statistical maps from all AEs were averaged (Fig. 3b). This revealed the highest accuracies in bilateral PTe and Te1.1/1.0 (green dashed line; left *n* = 323 voxels, right *n* = 267), as part of the VA. The highest decoding accuracies were found in Te1.0 and PTe, whereas Te3, which marks the center of the refined core VA (see Fig. 3b, left panel), shows moderate cross-classification results. Using this MVPA approach, we thus were able to define a subregion of the original VA that presumably decodes acoustic information that is common to voice sounds but also to several other AEs.

### Region-based analysis in functional and anatomical AC subregions

Besides its broad spatial extent in the AC, the VA was formerly proposed to host 3 distinct neural subpatches in both hemispheres located from posterior to anterior STC (Pernet et al. 2015). A detailed functional definition of these patches for voice processing is so far missing, but they might respond differently and more selectively to the presentation of AEs, compared to the original voices and nonvoices. Here, we took an approach based on a previous definition (Pernet et al. 2015) of the 3 patches in posterior, mid, and anterior STC (Fig. 3c) and defined these patches in each participant individually. Importantly, the original definition of these patches was not based on peaks of the averaged activation across participants but showed the most likely location of participant-specific peaks. We then performed the same MVPA analysis as described above on these ROI patches both in the left and right AC. This analysis was not performed as a searchlight analysis but on all voxels contained in the ROIs.

Figure 3c shows the cross-validation results within each sound set (i.e. original sounds, AEs) and the cross-classification (training on the originals sounds and testing on AEs) for the 3 VA patches (posterior, middle, and anterior) in both hemispheres. We tested for differences in classification accuracies for original sounds and for each AE cross-validation (panels “within”) and cross-classification accuracy from original sounds to AEs (panels “across”) using *t*-tests; each accuracy result was tested against chance-level, and we applied FDR correction to account for multiple testing. While all cross-validations of original sounds revealed above chance classification accuracies in all 3 patches, within the AEs this was only true for AE_e/s_ (all patches but left posterior VA patch) and the right anterior patch in the cross-validation of AE_scr_. The cross-classification approach only reveals above-chance classification accuracy for AE_e/s_ (in the right mid patch) and AE_scr_ (in the right anterior patch). The definition of these patches was based on a GLM contrast of voice versus nonvoice and might therefore bias the classification towards the differentiation of voices and nonvoices. This shortcoming was mitigated by performing the ROI-based analysis on anatomically defined subregions of the AC (Te1.0–1.2, PTe, and Te3) as an independent analysis from more functionally defined subareas of AC (Fig. 3d, FDR correction). A significant cross-validation approach for the original sounds was found in all anatomical subregions of the AC, except for left Te1.1. Significant cross-classification results were obtained for some of the AEs, such as AE_e/s_ (bilateral Te1.2 and Te3, right Te1.0–1.1 and PTe) and AE_scr_ (bilateral Te1.2 and Te3, right Te1.0). Significant cross-classification results were obtained for AE_e/s_ (bilateral Te1.2/1.0, Te3), AE_scr_ (bilateral Te1.2, right Te1.0, left Te3), and AE_p/a_ (bilateral Te1.2).

### The broader VA can be potentially subdivided into 3 functional subareas

**Fig. 4:**
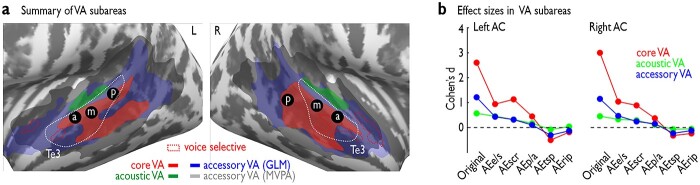
Summary of findings. a) Parcellation of the original VA into subfields based on their specificity for voice processing. The core VA (red) originating from the GLM interaction analysis (see Fig. 2b, green dashed line in the upper left panel) seems generic to voice processing since activity in these regions cannot be reduced to acoustic patterns in the comparison of voice and nonvoice sounds and their AEs. An acoustic VA (green) defined by the MVPA generalizations from original to AEs sounds (see Fig. 3b, green dashed line) seems to decode features that are relevant but not exclusive for voice from nonvoice discrimination. The accessory VA based on the GLM (blue; see Fig. 2a, black dashed line in left panels) and the MVPA approach (gray; see Fig. 3a, black dashed line) is only incidentally involved in voice processing. The dots denoted by a/m/p show the VA patches as previously defined (Pernet et al. 2015) that all uniformly are located within the core VA and that all seem to have a uniform function for generic voice processing. Red dashed line indicates potential voice-selective regions (see Fig. 2b). b) Effect size measures (Cohen’s *d*) for the 3 bilateral VA subareas (core VA, acoustic VA, accessory VA) in AC based on the group effect for comparing voice sounds (or their AEs) against nonvoice sounds (or their AEs). The bilateral core VA shows a highly increased effect size (Cohen’s *d* > 2.6) for original voice against nonvoice processing, which most likely originates from the interaction contrast effects shown in Fig. 2b.

Based on the results of the GLM and the MVPA analysis, a potential subdivision of the broader VA emerges. The VA in its original definition (Fig. 1a, left panel) usually covers broad areas of the higher-order AC with a large spatial extension from anterior to posterior STC and partly also covering areas of primary and secondary AC. Based on the results from the GLM interaction analysis (Fig. 2b), a “core VA” seems this part of the original VA that is involved in voice processing in its most specific sense that cannot be reduced to any processing that is elicited by the AEs (Fig. 4a). The MVPA analysis on the other hand pointed to another subarea of the original VA that is mainly involved in decoding acoustic features of sounds both in original sounds and the AEs and could be potentially termed as “acoustic VA” for detecting common and distinctive sound patterns across sounds. The remaining subparts of the original VA that were not covered by the core VA and the acoustic were termed as “accessory VA” here as its voice processing functions seem rather unspecific.

To quantify the functional specificity of each of these 3 VA subareas in the left and right hemispheres, we finally quantified effect sizes for each subarea by calculating Cohen’s *d* based on the difference between the voice and nonvoice sounds for the original sound category as well as for every of the AEs. Mean beta estimates were extracted for all voxels in each subarea for each participant, and a group Cohen’s *d* was calculated (Fig. 4b). The effect size was the largest for the left and right core VA, and the effect size considerably dropped for the AEs in the core VA, which resembles the interaction effect from the GLM analysis (Fig. 2b). The effect sizes in left and right acoustic VA and accessory VA were much lower compared to the core VA, especially for the original sound category as well as the AEe/s and AEscr sounds. These effect size patterns highlight the notion that voice processing is most specific in the core VA, with some residual voice sensitivity in the accessory VA, and stronger sensitivity to acoustic sound patterns in the acoustic VA.

## Discussion

Previous studies (Pernet et al. 2015; Aglieri et al. 2018) provided a cortical description of the VA that included a large spatial extension across many AC subregions and a rather uniform functional voice specificity across all VA subareas, including the spatially more localized VA patches. In the present study, we set out to refine the cortical description of the VA and provide a differential functional definition of VA subareas that are potentially closer to the general working principles of the AC (Fig. 4a). To this end, our study used a set of AEs to investigate the functional distinction in the VA where 2 complementary analysis types in the form of a univariate and a multivariate analysis were performed.

First, based on the results of our univariate GLM approach and the effect size measures, we propose a “core VA” that seems closest to a description of a specialized cortical voice processing area and covers only ~30% of the cortical size of the original VA. This confined definition of the core VA resulted from the interaction analysis (Fig. 2b, top left panel) where contrasting original voice against nonvoice sounds elicited significantly larger activity than for the same contrast for all AEs (Fig. 4b). The core VA covered large parts of the higher-level auditory cortical field of Te3 and extended into posterior part of the STS (pSTS). The core VA thus seems to predominantly include cortical regions for voice processing on a perceptual level that is beyond a basic acoustic feature processing. However, there seem to be some exceptions to this functional profile as we will discuss below. Second, we propose a VA subfield that we termed “acoustic VA” and that covers subparts of Te1.2 and lateral PTe. The acoustic VA resulted from the multivariate analysis approach, where a model trained to separate AC patterns of voice from nonvoice sounds was able to predict neural patterns for voice and nonvoice synthesized sounds across all AEs, but mainly for AE_e/s_ and AE_scr_ (Fig. 3b). Thus, the model generalized from the original voices to AEs. Given this functional response profile of the acoustic VA, we assume that this VA subfield is involved in the acoustic analysis of sounds for decoding acoustic information that is non-exclusive to voice sounds and that might only subsequently help to specifically detect voice sounds. Third, we propose that the remaining cortical areas that are usually covered by the original VA do only incidentally process voice sounds with some residual voice sensitivity, and we termed this area “accessory VA.” This incidental VA extends more anteriorly and posteriorly from the core VA areas and covers areas in the anterior and posterior STC. These areas are rather associative cortical areas involved in the processing of higher-order information from various multimodal social signals but presumably also from voice sounds (Perrodin et al. 2015; Tsantani et al. 2019).

Voice signals are specific and important acoustic objects for human social interactions, and the existence of a specialized brain area for voice processing seems intuitive. Yet, the present study suggests that the cortical spatial extent of the original VA might have been largely overestimated and that only a small part of the VA (~30%) might be termed to be generically involved in voice processing. We termed this refined area as the “core VA” as it showed higher activity for voice sounds beyond any acoustically similar sounds. This centrally located part of bilateral Te3 seems to be involved in the processing and discrimination of auditory objects (Leaver and Rauschecker 2010) and especially of voice sounds as a specific type of auditory object (Perrodin et al. 2011; Bizley and Cohen 2013). The Te3 also functions as a region for voice processing that is downstream to the processing of more basic acoustic features that are characteristic of voice sounds (Lewis et al. 2009; Leaver and Rauschecker 2010). Given these results, the area of the core VA inside Te3 seemed like a generic cortical voice processing field.

This core VA could potentially represent a “voice-selective” subarea of the AC as proposed in previous studies (Belin et al. 2000; Pernet et al. 2015). We tested for such voice-selectivity in the AC and especially in the broader VA by determining the response magnitude for original voice sounds across voxels and compared the response magnitude for all AEs with the original voice sounds. A voxel was classified as voice-selective if none of the magnitudes for all AEs was higher than 33%. This would mean that a region shows activity for voice sounds that is several times higher than for any other sound presented here. A first finding was that none of the voxels showed consistent voice-selectivity across all participants. Although we found regions of voice-selectivity in some AC voxels in each participant, there was reduced spatial overlap of the regions, with the highest consistency of *n* = 13 out of the total 25 participants (Supplementary Fig. S2b). These regions with the highest probability of voice selective voxels across participants were located in bilateral PPo as part of secondary AC as well as in anterior STC as part of higher-order AC in Te3. The latter region in anterior STC partly overlapped with the region that we defined as core VA, which could point to the notion that part of the core VA is indeed voice-selective, but with the limitation that voice-selectivity was only at a small-to-medium level in our study. The potential voice-selective region in PPo was located outside our core VA. The PPo is a relatively unexplored area in sound and especially voice processing (Warren et al. 2003; Kim and Knösche 2016), but it was located within our broader accessory VA. The PPo is sensitive to musical sounds, probably based on decoding pitch chroma in sounds (Kim and Knösche 2016), which is also an important feature for voice sound detection and discrimination (Latinus and Belin 2011). The core VA might therefore be extended to PPo, potentially ranging into an anterior voice patch in anterior STC as a central cortical voice processing node (Pernet et al. 2015; Belin et al. 2018). The exact functional role of the PPo in voice processing and its precise level of voice-selective however might have to be determined in future studies, also including a cross-comparison to animal data (Petkov et al. 2008).

The results that we obtained with the multivoxel analysis indicated that the other parts of Te3 are nevertheless also involved in some type of acoustic analysis of sound patterns, especially in the left AC. This overlaps anatomically with the mid and anterior VA patches (Pernet et al. 2015), which would point to the notion that there is some functional processing difference between the VA patches. When we trained a classifier to separate neural patterns of voice from nonvoice sounds, we first found high decoding accuracies for separating the original sounds in mid and anterior VA patch as well as in Te3. This is indicative of a specialization for voice processing in these regions. Importantly, the VA patches were identified by a voice/nonvoice contrast, which renders them nonindependent from decoding accuracies by potentially inflating the voice versus nonvoice classification accuracy, but the analysis provides a direct comparison with earlier studies. Second, using this trained classifier to also predict neural pattern discriminations for the AEs, we found significant discrimination accuracies in Te3 and left mid VA patch for AE_e/s_ sounds as well as in left Te3 and anterior VA for AE_scr_ sounds. This activation pattern was also confirmed by the simple contrasts between the AE-voice and AE-nonvoice sounds for the AE_e/s_ and AE_scr_ categories (Fig. 2a). Thus, the data of our MVPA analysis point to some differential acoustic representation in left VA patches that might support voice discrimination. A neural cross-classification from original sounds to AE_e/s_ (preserving the global spectrotemporal profile of original sounds) was possible in the left mid VA patch, while this cross-classification to AE_scr_ (preserving spectral content) was possible in the left anterior VA patch. This suggests that different VA patches might represent form and content of spectral information that is necessary to discriminate voices from nonvoices (Moerel et al. 2013; Norman-Haignere et al. 2015).

The AE_e/s_ and AE_scr_ were the AEs that preserved most of the acoustic information of the original sounds, including the perceptual impression of a “voice-similarity” of the sounds, as visible from the results of perceptual ratings (Fig. 1b). This acoustic information might thus serve to generate a first but insufficient prediction of a sound as potentially originating from a voice source, and this first prediction might then need to be confirmed by further neural processing (Pachitariu et al. 2015; Heilbron and Chait 2018). This final confirmation for this first prediction could be provided by processes in other dedicated subareas of Te3, but potentially also by the pSTS as the second major and potentially downstream subfield covered by the core VA (Rauschecker and Scott 2009).

For the posterior part of the core VA, the MVPA showed significant decoding accuracies for separating the original voice from nonvoice sounds, but no transfer of this information to separate these categories for the AE in the cross-classification approach. The pSTS thus might be indeed involved in a generic voice sound detection. The pSTS is relevant for classifying auditory objects (Liebenthal et al. 2010) and auditory communication signals (Shultz et al. 2012) but also for many subfunctions of social cognition (Deen et al. 2015) that are also relevant for the classification of and the social cognition from voices (Kriegstein and Giraud 2004). The pSTS also includes fields for multisensory processing of social information from voices accompanied by relevant information on other sensory modalities (Kreifelts et al. 2007; Chandrasekaran and Ghazanfar 2009). The part of the core VA located in the pSTS might therefore classify sounds as voice signals but might also propagate this information for further detailed processing of voice information and social analysis. To summarize the findings on the core VA, we could confirm that this brain region seems specialized for voice processing beyond decoding basic acoustic differences of voice and nonvoice sounds as previously observed (Agus et al. 2017). However, while these authors (Agus et al. 2017) found that the original VA still reliably distinguishes voices from nonvoices when controlling for acoustic features (pitch, HNR, temporal rate, and spectral scale) and propose the VA to be specific to voices beyond acoustic feature analysis, our core VA is smaller than the original VA and is assisted by a distinctive acoustic VA and accessory voice fields.

Adjacent to the core VA that covered a part of the original VA, we identified an area that we termed acoustic VA that was located superior to the core VA at the intersection of Te1.0/1.2 and lateral PTe. The acoustic VA covered 16.9%–19.5% of the original VA. We termed this VA subfield as acoustic VA, because the MVPA analysis showed that a classification model using information from neural patterns for separating voice from nonvoice sounds was able to separate the same categories in the AEs that preserved some important acoustic features and patterns of the original sounds. The acoustic VA thus seems to analyze acoustic information that is contained in, but is not exclusive to, voice sounds. Interestingly, when we performed the MVPA analysis on anatomical subregions of AC, no significant cross-classification results were found for the entire region of the PTe. Although neural patterns for original voice and nonvoice sounds were discriminative in bilateral PTe, this information did not generalize to the AEs in the cross-classification approach in PTe. While this might rather indicate that there is no acoustic information shared between original sounds and the AEs to elicit similar neural patterns in these regions that were underlying the acoustic VA, we have to note that the acoustic VA only covered a small part of these anatomical regions, especially in lateral and anterior PTe. PTe has been associated with early auditory processing and showed similar activity for listening to nonvoices (tones) and voices (words) (Binder et al. 1996). Unlike the medial PTe, the lateral part of PTe seems indeed more sensitive to simple acoustic sound patterns than for complex auditory objects (Griffiths and Warren 2002), and anterior PTe also seems sensitive to pitch changes in sounds (Warren and Griffiths 2003) as a potential relevant cue for voice signal detection. Unlike for the PTe, the lateral part of primary AC on Te1.2 showed extensive cross-classification results, also with AEs (i.e. AE_p/a_) that were perceptually quite distant from the original sounds, which strongly qualifies this region as an acoustic VA.

The core VA and the acoustic VA described above covered parts of the original VA. We termed the remaining cortical area of the original VA that was not covered by the core and the acoustic VA as the accessory VA. The accessory VA is voice sensitive given its higher neural activity when comparing original voices against nonvoices, but it does not seem to have a central and mandatory functional role in voice processing as compared to the core and acoustic VA. The accessory VA was covering the remaining 47.4%–52.7% of the original VA and extended into anterior and posterior STC. These anterior and posterior regions of the VA were also the cortical areas that could not be explained by original voice processing in the interaction analysis against any of the AEs. Even for the AEs that were most distant to the original sounds (AE_p/a_, AE_rip_, and AE_tsp_), there were some parts, especially in anterior STC and the PPo, for which the original voices did not reveal higher activity than the AEs. The PPo is associated with many sound processing functions, such as pitch chroma (Warren et al. 2003) and melodic aspects of human vocalizations (Angulo-Perkins and Concha 2019). Unlike the PPo, the very anterior and posterior regions of STC are assumed to be higher-order association areas, especially for the multimodal integration of social information (Kreifelts et al. 2007; Chandrasekaran and Ghazanfar 2009) and for further extraction of social and potentially linguistic information from voice signals after the stage of voice detection (Milesi et al. 2014; Perrodin et al. 2015).

Referring to this general level of processing voice signals, voices can be discriminated from nonvoices not only on their perceptual and neural levels as described above, but they might also critically differ in their general acoustic patterns. We therefore finally applied a similar SVM decoding analysis as we used for the neural data to the acoustic profile of voice and nonvoice sounds. Using a cross-validation approach to separate original voices from nonvoices based on 88 central voice features, we revealed the highest discrimination accuracy for the original sounds compared to the same procedure applied to each of the AEs. For the AEs, the AE_scr_ revealed the highest discrimination accuracy, followed by AE_p/a_ and AE_e/s_. This order of discriminability did not exactly follow the order of neural effects of the AEs, where AE_e/s_ revealed the strongest neural effects close to the original sounds, followed by AE_scr_ and AE_p/a_. Thus, there seemed to be no large match between the level of the acoustic discriminability of the AEs and their neural signature. While a neural association with acoustic pattern discrimination in primary AC is a common observation (Kumar et al. 2014), the Te3 was the region with broad coverage of the core VA that prima facie seemed the most distant from the acoustic pattern discrimination level but did include some processing properties for complex spectral patterns inherent to sounds and specifically voices.

Given this acoustic pattern sensitivity in Te3, a final notion concerns the differentiation into 3 bilaterally symmetric VA patches by previous studies (Pernet et al. 2015; Belin et al. 2018). Although this differentiation into separate VA patches would point to a functional differentiation inside the VA, investigating the functional properties of the VA patches is so far missing. Given that in our study all VA patches were located inside the core VA with a rather uniform function of discriminating voice from nonvoice sounds at a high level of neural abstraction, a clearer functional description and functional differentiation of the VA patches seem warranted. However, the data of our multivariate analysis point to some differential acoustic representation in left VA patches that might support voice discrimination. A neural cross-classification from original sounds to AE_e/s_ (preserving the global spectrotemporal profile of original sounds) was possible in the left mid VA patch, while this cross-classification to AE_scr_ (preserving spectral content) was possible in the left anterior VA patch. Thus, different VA patches might represent form and content of spectral information that is necessary to discriminate voices from nonvoices (Moerel et al. 2013; Norman-Haignere et al. 2015).

Finally, our study comes with 3 potential limitations. First, the original nonvocal sounds were more similar to their AEs as original vocal sounds to their respective AEs according to the perceptual ratings results. It could be that the voice signals contain certain acoustic features to a greater extent than the nonvoice sounds, which reduces the similarity to their AEs when only preserving these acoustic features. The creation of AEs only preserving certain acoustic features thus might have differentially affected vocal and nonvocal sounds. However, this notion and potential limitation seem rather implausible, as in our analysis no activation in certain regions showed higher activity by contrasting AEs to their original sounds ([AEvoice > AEnonvoice] minus [ORIGvoice>ORIGnonvoice]). Second, we could show that envelope and spectrum preserved AEs as well as scrambled sounds seemed to carry acoustic information relevant for the brain to distinguish voice from nonvoice sounds, which was possible to a lesser degree only for AE_p/a_, AE_rip_, and AE_tsp_. These latter AEs appeared to be less similar to the original sounds (perceptual ratings), giving credence to the above notion of a differential relevance for certain sound features. However, we cannot rule out that other not investigated acoustic features might be represented by subareas of the VA, especially the accessory parts of the original VA. Furthermore, as the refined core VA, which represents voice processing specificity beyond sensitivity to certain acoustic features, is spatially much smaller than the original VA, the accessory areas might indeed process acoustic information of AE_p/a_, AE_rip_, and AE_tsp_. But this acoustic information might not be sufficient to discriminate between voice and nonvoice sounds. Third and finally, the original set of stimuli contained 29 speech and 41 nonspeech stimuli for the category of vocal stimuli. Previous studies have shown that speech sometimes elicit larger AC activity than nonspeech vocal stimuli (Norman-Haignere et al. 2015; Deen et al. 2020). Some of the neural effects of processing voices and their AEs reported here might therefore be driven more strongly by processing speech rather than nonspeech vocal sounds and need to be further explored in future studies. However, most of the speech stimuli used here used speech material of rather basic linguistic structure (i.e. simple words or interjections in a foreign language) that elicit only basic speech-specific processing dynamics compared to complex words and sentences.

## Supplementary Material

StaibFruhholz_CerCor2022_supplement_bhac128
