# Supplementary material for: Distinct functional levels of human voice processing in the auditory cortex
Source: Cereb Cortex. 2022 Mar 26;33(4):1170–85. doi: 10.1093/cercor/bhac128 (PMC9930621; doi:10.1093/cercor/bhac128)

Supplemental material
Distinct functional levels of human voice processing in the auditory cortex
Matthias Staib, Sascha Frühholz

Supplemental figures

Figure S1. Replication of neural activations analyses with a smoothing kernel of 4mm^3 isotropic smoothing. (a) Replication of the analysis as reported in Fig. 2a (left panel). (b) Replication of the analysis as shown in 2b (left upper panel).

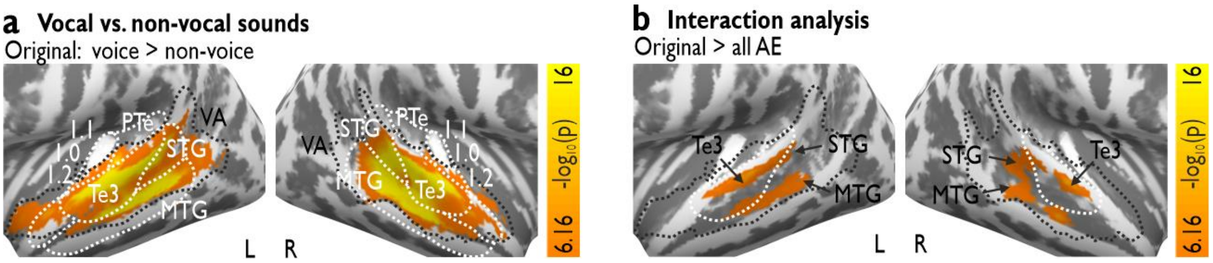

Figure S2. Quantification of response magnitude and voice-selectivity. (a) Voxel-wise voice-selectivity across participants. The map shows the consistence of voice-selectivity across participants, ranging from 1 to 25 (n=25 is the potential maximum given the sample size). (b) Same map as in (a), but threshold to show voxels for which consistency was found for at least n=11 participants; maximum number of participants showing consistency in the maximum voxel was n=13, no voxel showed higher values.

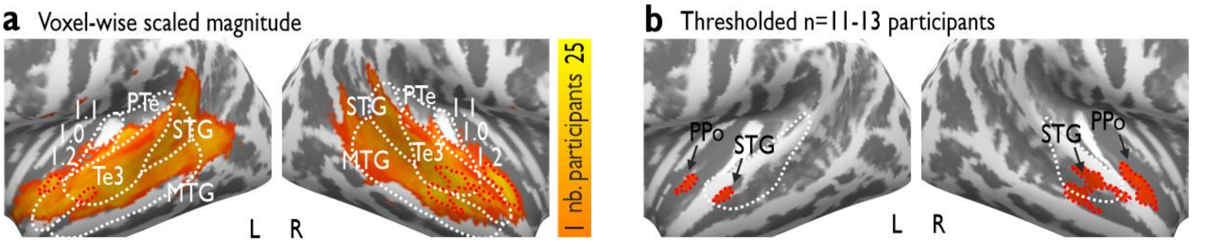

Supplement: StaibFruhholz_CerCor2022_supplement_bhac128 [file staibfruhholz_cercor2022_supplement_bhac128.pdf]
